# Iron in Hydroxyapatite: Interstitial or Substitution Sites?

**DOI:** 10.3390/nano11112978

**Published:** 2021-11-05

**Authors:** Leon Avakyan, Ekaterina Paramonova, Vladimir Bystrov, José Coutinho, Sandrine Gomes, Guillaume Renaudin

**Affiliations:** 1 Faculty of Physics, Southern Federal University, 5 Zorge St., 344090 Rostov-on-Don, Russia; 2 Institute of Mathematical Problems of Biology, Branch of Keldysh Institute of Applied Mathematics, Russian Academy of Sciences, 1 Vitkevicha St., Pushchino, 142290 Moscow, Russia; ekatp11@gmail.com (E.P.); vsbys@mail.ru (V.B.); 3 I3N, Department of Physics, Campus Santiago, University of Aveiro, 3810-193 Aveiro, Portugal; jose.coutinho@ua.pt; 4 Université Clermont Auvergne, Clermont Auvergne INP, CNRS, ICCF, F-63000 Clermont-Ferrand, France; sandrine.renaudin@sigma-clermont.fr (S.G.); guillaume.renaudin@sigma-clermont.fr (G.R.)

**Keywords:** iron doping, hydroxyapatite bioceramics, density functional theory, hybrid density functional, X-ray absorption spectroscopy

## Abstract

Iron-doped hydroxyapatite (Fe-HAp) is regarded as a promising magnetic material with innate biocompatibility. Despite the many studies reported in the literature, a detailed theoretical description of Fe inclusions is still missing. There is even no consensual view on what kind of Fe defects take place in Fe-HAp—iron interstitial or calcium substitutions? In order to address these questions, we employ modern first-principles methodologies, including hybrid density functional theory, to find the geometry, electronic, magnetic and thermodynamic properties of iron impurities in Fe-HAp. We consider a total of 26 defect configurations, including substitutional (phosphorus and calcium sites) and interstitial defects. Formation energies are estimated considering the boundaries of chemical potentials in stable hydroxyapatite. We show that the most probable defect configurations are: Fe${}^{3+}$ and Fe${}^{2+}$ substitutions of Ca(I) and Ca(II) sites under Ca-poor conditions. Conversely, Fe interstitials near the edge of the hydroxyl channel are favored in Ca-rich material. Substitutional Fe on the P site is also a probable defect, and unlike the other forms of Fe, it adopts a low-spin state. The analysis of Fe K-XANES spectra available in the literature shows that Fe-HAp usually contains iron in different configurations.

## 1. Introduction

Ceramic materials based on calcium apatites are important substances in medicine, biology, ecology and catalysis [1,2,3,4]. Among the apatites, hydroxyapatite (Ca${}_{10}$(PO${}_{4}{)}_{6}($OH${)}_{2}$ = HAp) is singled out as the main inorganic component of vertebrate bones and teeth. Biogenic HAp differs from the synthetic compound due to the presence of foreign atomic (Mg${}^{2+}$, Na${}^{+}$, Cl${}^{-}$) and molecular (CO${}_{3}^{2-}$, SiO${}_{4}^{4-}$) ions. Although the role of most impurities is largely unknown, some studies suggest that the presence of a small concentration of iron (below 157 ppm) prevents the loss of calcium from bones and desorption of enamel from teeth [5,6]. Iron-doped HAp (Fe-HAp) has also attracted interest due to its intrinsic magnetic properties [7]. It is a promising material for magneto-resonance imaging agents [8], heat centers for magnetic hyperthermia [4,9], sunscreen filter creams [10], antimicrobial coatings [11], and to be incorporated in drug delivery systems [12,13].

Despite nearly five decades of research on Fe-HAp and related systems [6,14,15,16,17,18,19], our knowledge regarding the atomistic and electronic structure and iron defects is still very limited. The main difficulty arises from the quality of the samples—single-phase products are hard to synthesize, iron metal and iron oxide often segregate at the surface, and the admixture of stable phosphates, in particular tricalcium phosphate (Ca${}_{3}$(PO${}_{4}{)}_{2}$ = TCP), is usually observed [16,18]. Discrepancies between the results from different authors are often attributed to different preparation conditions, including sintering temperature and atmosphere, the duration and temperature of doping treatments, the source of precursors, etc. A comprehensive review of experimental methods and results can be found in Ref. [6].

**Figure 1:**
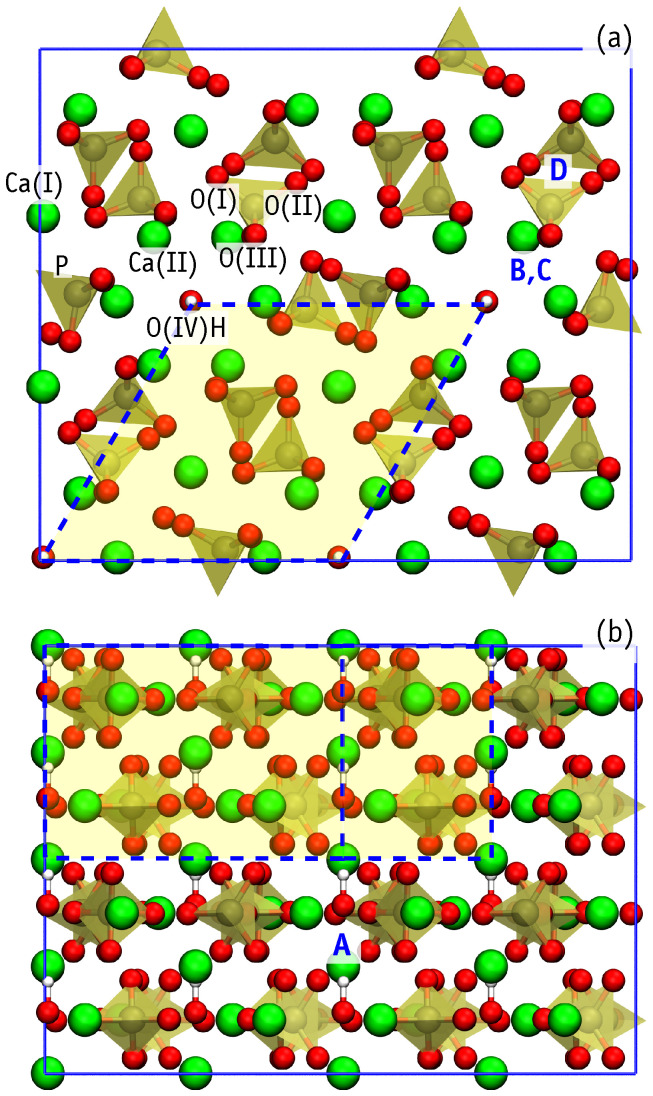
Top-projection (**a**) and side-view (**b**) of an orthorhombic 2×2×2 HAp supercell with the hexagonal unit cell highlighted. O, Ca and H atoms are shown in red, green and white, respectively. Each PO${}_{4}$ group contains O(I), O(II) and two O(III) sites. Capital letters A-D indicate the location of the interstitial sites considered.

The HAp structure contains two symmetrically distinct positions of calcium, Ca(I) and Ca(II) (Figure 1), both of which are candidates for iron replacement. The pioneering study of Fe-HAp using Mössbauer (MB) spectroscopy by Ok [14] indicated the preference of Fe${}_{\mathrm{Ca}(I)}$ over Fe${}_{\mathrm{Ca}(\mathrm{II})}$, while subsequent MB studies [15,16] concluded on comparable amounts of Fe${}_{\mathrm{Ca}(I)}$ and Fe${}_{\mathrm{Ca}(\mathrm{II})}$ defects. In both cases, electron paramagnetic resonance (EPR) data indicated the presence of isolated high-spin Fe${}^{3+}$ ions with 4 or 6 neighbors, in coexistence with superparamagnetic iron oxide on the surface. The MB experiments of Boda et al. [6] suggest that ${\mathrm{Fe}}_{\mathrm{Ca}(I)}^{3+}$ defects are more probable when conventional sintering is used during sample preparation, whereas ${\mathrm{Fe}}_{\mathrm{Ca}(\mathrm{II})}^{3+}$ substitutions appear as a result of hot pressing.

Interstitial iron (Fe${}_{i}$) defects have been reported as well. Analysis of X-ray diffraction (XRD) data shows that iron can occupy 2b or 12i Wyckoff positions [5,18,19,20]. The first site corresponds to the formation of a linear O-Fe-O structure in the center of the OH channel (site A at Figure 1), while the latter involves a three-fold coordinated Fe atom displacement towards the wall of the channel (site C at Figure 1). However, from XRD and Raman scattering data, Kato et al. [19] argues on the formation of four-fold coordinated Fe. The absence of Fe${}_{\mathrm{Ca}}$ in these experiments was postulated based on the lack of CO${}_{3}^{2-}$ groups [6,16]. These are commonly present in biogenic HAp, and lead to the formation of several defects, including: [CO${}_{3}^{2-}$]${}_{\left[{\mathrm{PO}}_{4}^{3-}\right]}$, [CO${}_{3}^{2-}$]${}_{\left[{\mathrm{OH}}^{-}\right]}$, [O${}^{2-}$]${}_{\left[{\mathrm{OH}}^{-}\right]}$ and, finally, [Fe${}^{3+}$]${}_{{\mathrm{Ca}}^{2+}}$. Here we limit our analysis to iron-only defects, leaving carbonate-related defects outside the scope of this work.

Besides the lack of agreement between different MB studies, XRD data also holds controversy due to a low electronic contrast between Fe${}^{3+}$ and Ca${}^{2+}$ ions [18]. Hence, atomic-level simulations are regarded as a source of clarification. Using empiric interatomic potentials, Jiang et al. [15] concluded that the Fe${}^{2+}$ ion finds its favorite site at the Ca(I) position, while Fe${}^{3+}$ favors the Ca(II) site. More recently, Zilm et al. [17] investigated this problem using semi-local density functional theory, but the results were limited to the 2+ oxidation state. Despite enlightening, both studies focused on Fe${}_{\mathrm{Ca}}$ substitutions only, with alternative substitutional and interstitial forms of iron being overlooked. Hence, we may conclude that the starting point of the present work is a largely unknown picture of Fe impurities in HAp regarding their local structure, oxidation and magnetic states, and optoelectronic properties.

Recently we demonstrated [21] that the use of hybrid density functional theory (hybrid-DFT) can describe the electronic properties of HAp with an accuracy that rivals with highly accurate Green’s function (*G*) method with screened interaction (*W*), so called GW approximation [22]. The GW and hybrid-DFT methods provided band gaps of HAp close to 7 eV, but generalized gradient functional resulted in a 30 % smaller gap [23,24,25,26,27,28]. Errors of the same magnitude are therefore expected for the calculated optoelectronic properties of defects in HAp, as shown for the case of the OH vacancy [21].

In view of the above, we present a hybrid-DFT study of the most probable Fe-related defects in hydroxyapatite (substitutional and interstitial). The results are interpreted and scrutinized from the perspective of the available experimental data. The paper is organized as follows: Section 2 outlines the methodologies and computational details. Section 3 discusses the results, subdivided in Subsections pertaining the chemical phase diagram of HAp (Section 3.1), the structure and thermodynamics of neutral and charged defects (Section 3.3 and Section 3.4, respectively), and a simulation of the Fe K-XANES spectra of Fe-HAp available in the literature (Section 3.5). Finally, Section 4 summarizes the results.

## 2. Computation Methods

HAp crystallizes in the form of an molecular ionic crystal of symmetry $P6_{3}/m$ (#176 in the crystallographic tables), whose hexagonal unit cell encloses two Ca${}_{5}($PO${}_{4}{)}_{3}$OH units [29,30]. Figure 1 illustrates the supercell used in this study, comprising 8 unit cells (16 HAp formula units). HAp primitive cell contains two and four inequivalent calcium and oxygen sites, respectively. The Ca(I) cation columns are surrounded by O(I) and O(II) from PO${}_{4}$ anion groups, while mirror-symmetric O(III) sites and Ca(II) ions form a hexagonal channel enclosing the hydroxyl anions O(IV)H. Variation of alignment of OH dipoles in these channels lead to different HAp phases. Namely: (i) a hexagonal disordered phase, with random orientations of OH dipoles; (ii) a hexagonal ordered phases, where OH dipoles are all oriented along the same direction, or (iii) a monoclinic ($P2_{1}/b$) phase, made of two cells repeated along a basal lattice vector (a or b), with the first possessing a ...-OH-OH-... column, while the second one showing an opposite ...-HO-HO-... ordering [31]. The last phase shows anti-ferroelectric properties [32]. Given that the electronic band structure of the above polytypes is rather similar [27], the impact of OH-flipping on the problems being investigated is expected to be minor. Henceforth, we will consider the introduction of iron defects in the ordered hexagonal phase.

### 2.1. First-Principles Defect Energetics

The calculations were performed using density functional theory using the QUANTUM ESPRESSO package [33,34]. The many-body electronic potential was evaluated using the hybrid density functional of Heyd, Scuseria and Ernzerhof (HSE) [35,36]. The Kohn-Sham eigenstates obtained using PBE [37] exchange-correlation functional were used to initiate the self-consistent cycle. Due to severe limitations of the semi-local functionals (such as PBE) in describing the electronic structure of HAp in the band gap vicinity [21], the application of hybrid-DFT turns out to be critical in the evaluation of the stability of charged defects.

By opting for a local treatment of the electronic correlation, we expect some error in the description of the d-shell in Fe. Using a GGA+U approach would lead to a narrow gap material, and hence to an artificial hybridization between the d-levels of Fe and those from the conduction and valence band edges of HAp. Unfortunately, it was not possible to combine the non-local HSE functional, with the Hubbard-like local correction to the electronic correlation on the Fe atom.

Core electron states were described by means of optimized norm-conserving Vanderbilt pseudopotentials [38,39], while the Kohn-Sham problem was solved within the plane wave formalism with cut-off energy up to 60 and 240 Ry to expand the wave functions and semi-local potential. The exact exchange operator was evaluated on a grid which corresponded to a plane wave cut-off of 120 Ry. The occupation of states in the vicinity of the Fermi energy were smeared out with a Gaussian function of width 0.002 Ry to improve convergence.

The equilibrium lattice parameters of the unit cell were obtained a=9.481 Å and c=6.859 Å using HSE exchange-correlation functional. These values are particularly close to a=9.417 Å and c=6.875 Å from an experimental report [30].

Defects were introduced in orthorhombic supercells made up of 8 HAp primitive cells (spacegroup $P6_{3}/m$), containing a total of 352 atoms. The smooth dispersion of the band structure of the large HAp supercell allowed us to use a single point (k=Γ) for sampling the Brillouin zone (BZ). Convergence tests showed that the total energy of such supercells obtained with Γ only sampling, differs by less than 0.1 eV (0.3 meV/atom) from a calculation where the zone was sampled over a 2×2×2 grid of k-points.

Defect containing supercells were first relaxed on a PBE level, since claculation of forces on a hybrid-DFT level is prohibitively expensive for large systems. During the relaxations, coordinates of all atoms were varied until the maximum force became less than 0.4 mRy/Bohr ≈10 meV/Å. On a second step the total energy was calculated on hybrid-DFT level for obtained structures. It was shown [40,41,42] that relative errors in energies obtained within this methodology usually have an order of 10 meV.

The formation energy $E_{f}$ of a defect in a crystalline sample can be expressed as [43]:

(1) $$E_{f}=E_{d}\left(q,R\right)-E_{\mathrm{HAp}}-\sum_{i}\Delta n_{i}\mu_{i}+q\left(E_{v}+E_{F}\right),$$

where the main contribution is the energy difference between defective ($E_{d}$) and pristine ($E_{\mathrm{HAp}}$) crystals. *R* and *q* denotes the defect configuration and charge state, correspondingly. Last two terms account for stoichiometric and charge differences between the defect and pristine cells. In particular, $\mu_{i}$ is the chemical potential of species *i* which must be added ($\Delta n_{i}$ > 0) or removed ($\Delta n_{i}$ < 0) to or from the ideal crystal to create the defect, respectively. The last term in Equation (1) accounts for the exchange of electrons between the defect with charge *q* and an *electron reservoir* with chemical potential $\mu_{e}$ = $E_{v}$ + $E_{F}$. Here $E_{v}$ and $E_{F}$ denotes the energy of the valence band top and Fermi level, respectively. The Fermi energy can vary within the band gap ($0\leq E_{F}\leq E_{g}$), where $E_{g}$ is the band gap width), depending on the doping of the crystal.

The variation of chemical potentials $\mu_{i}$ is also limited,

(2) $$\sum_{i}n_{i}^{\phi }\cdot \left(\mu_{i}-\mu_{i}^{0}\right)\leq \Delta H_{f}^{\phi },$$

with the upper limit taking place if HAp becomes unstable with respect to formation of a compound ϕ made of $n_{i}^{\phi }$ elements of species *i* and with heat of formation $\Delta H_{f}^{\phi }$. The chemical potentials of O, H, Ca and P in standard phases, $\mu_{i}^{0}$, were calculated from the energy per atom in molecular oxygen, molecular hydrogen, calcium metal and black phosphorous. The molecules (O${}_{2}$ in the spin-triplet state or H${}_{2}$) were placed in a simulation cubic box of 20 Å size. The chemical potential of iron was found from the *bcc-*Fe phase with the BZ sampled over a 16×16×16 k-point mesh. The resulting ground state spin density corresponded to a magnetic moment of 2.80 $\mu_{B}$ per cell. The deviation from the experimental value of 2.22 *μ_B_* results from a known over-localization of the exchange interactions in bulk Fe when using hybrid functionals [44]. An identical result was obtained using projected augmented-wave potentials. Nevertheless, it is noted that hybrid functionals have successfully been used for the study of iron oxides and defects in oxides (see for instance [45,46]).

The heat of formation of the compounds ϕ used in Equation (2) was estimated from their hybrid-DFT total energies ($E^{\phi }$) with respect to energies of their constituents in their standard phases ($\mu_{i}^{0}$),

(3) $$\Delta H_{f}^{\phi }=E^{\phi }-\sum_{i}n_{i}^{\phi }\mu_{i}^{0}.$$

Due to periodic boundary conditions, the calculation of charged defects (q≠0) is accompanied by a compensating uniform charge density of opposite sign [47]. The artificial interactions between the periodically repeated charged defects and the background lead to the deviation of calculated periodic total energy ${\tilde{E}}_{d}$ from $E_{d}$, in Equation (1). The energy correction $E_{\mathrm{corr}}$, so that $E_{d}={\tilde{E}}_{d}+E_{\mathrm{corr}}$, is obtain following the recipe of Lany and Zunger [48],

(4) $$E_{\mathrm{corr}}\approx \frac{2}{3}\frac{\alpha_{M}q^{2}}{\epsilon \;L},$$

where $\alpha_{M}$ is the Madelung constant of the HAp supercell with edge length *L*, and ϵ is the static dielectric constant (ϵ≈11 [21]). For a singly charged defect the correction amounts to about 0.04 eV. However, this figure can grow considerably in the case of multi-ionized defects ($E_{\mathrm{corr}}\approx 0.33$ eV for q=+3).

The magnetization of the Fe defects was calculated from spin-polarized electron densities $n_{↑}\left(r\right)$ and $n_{↓}\left(r\right)$,

(5) $$M_{z}=\int_{\Omega }\left(n_{↑}-n_{↓}\right)\;d^{3}r,$$

while formal oxidation states of iron ions were deduced from the occupations of d-orbitals as proposed by Sit et al. [49].

### 2.2. X-ray Absorption near Edge Structure (XANES)

In order to compare the defect structures found from the first-principles total energy calculations, with those reported in the literature, we simulated the Fe K-XANES spectra for each structure. The calculations were performed using a full-multiple scattering method and the “muffin-tin” approximation for the interatomic potential as implemented in the FDMNES code [50]. The size of the atomic cluster and spectral convolution parameters were adjusted using the spectra of magnetite Fe${}_{3}$O${}_{4}$ as reference. The radius of the cluster was estimated of 7 Å which gives cluster of Fe${}_{1}$Ca${}_{∼30}$P${}_{∼15}$O${}_{∼70}$H${}_{∼5}$, with exact composition dependent on the structure. The used approach cannot reproduce pre-edge features, which is a well known limitation of a description based on dipolar transitions and the single-particle approximation [51,52].

## 3. Results and Discussion

### 3.1. Chemical Stability Diagram

The formation of the most probable iron-related defects in HAp depends most notably on the chemical potentials of the several elements involved. Each chemical potential can vary within a certain range, limited by thermodynamic stability conditions of the HAp crystal itself. We estimate the ranges using Equation (2) with respect to a set of boundary phases {ϕ}. The methodology used to find the structure and energy of each phase was identical to that used for the HAp supercell. This includes the exchange-correlation functional and energy cutoffs. The first candidates for the bordering phases are those involved in the HAp synthesis. There is a variety of production routes, but we will only consider reactants whose species are common to those found in HAp, namely CaO [6], Ca(OH)${}_{2}$ [8], H${}_{3}$PO${}_{4}$, H${}_{2}$O and P${}_{2}$O${}_{5}$ [18]. Next, we considered calcium phosphates: dicalcium phosphate dihydrate CaHPO${}_{4}\cdot 2$H${}_{2}$O = DCPD (mineral brushite), anhydrous dicalcium phosphate CaHPO${}_{4}$ = DCPA (mineral monetite), tricalcium phosphate Ca${}_{3}$(PO${}_{4}{)}_{2}$ = TCP and tetracalcium phosphate Ca${}_{4}$(PO${}_{4}$)${}_{2}$O = TTCP. The last two have [Ca]: [P] concentration ratio of 1.5 and 2.0, respectively, while HAp has an intermediate [Ca]: [P] ratio of 5/3≈1.67.

Table 1 presents the formation energies $\Delta H_{f}$, calculated according to Equation (3) within HSE, for the materials enumerated above. The overall agreement with the reference data [53,54] (including the energy, cell volume and bulk modulus) is in line with the usual accuracy of hybrid-DFT. The phosphoric acid and water were calculated in a gas phase (single molecules in large periodic box), hence the lack of cell volume and bulk modulus.

From the data of Table 1 and Equation (2), we can estimate the chemical potential ranges within which HAp is thermodynamically stable. We can also reduce the number of independent chemical potentials, for instance to $\mu_{H}$, $\mu_{\mathrm{Ca}}$ and $\mu_{P}$, by expressing the chemical potential of oxygen as a function of $\mu_{i}$ of the remaining elements (c.f. Equation (2)),

$$\mu_{O}-\mu_{O}^{0}=\left[\Delta H_{f}^{\mathrm{HAp}}-\sum_{i=\mathrm{Ca},\;P,\;H}n_{i}^{\mathrm{HAp}}\;\left(\mu_{i}-\mu_{i}^{0}\right)\right]/n_{O}^{\mathrm{HAp}}.$$

Let us look first at the range of hydrogen chemical potentials. In this case, phases with stoichiometry (not) satisfying $n_{H}^{\phi }\cdot n_{O}^{\mathrm{HAp}}<n_{O}^{\phi }\cdot n_{H}^{\mathrm{HAp}}$ give lower (upper) bounds for $\mu_{H}$. In particular, the planes corresponding to CaO, TCP and pure O${}_{2}$ may form lower bounds, while planes for hydrogen-containing phases may be responsible for upper bounds.

**Figure 2:**
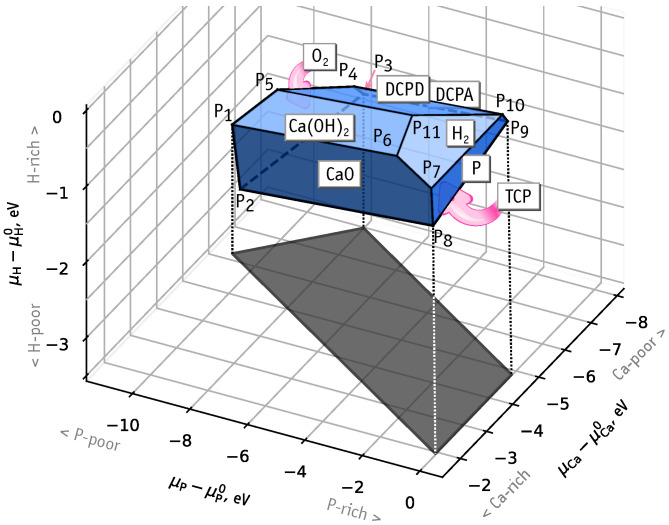
Domain of chemical stability of HAp represented by the limits of chemical potentials of Ca, P, H. Symbols $P_{1}–P_{11}$ correspond to points where three boundary phases coexists with the HAp crystal.

According to our calculations, the HAp stability domain is limited by CaO, Ca(OH${)}_{2}$, P, H${}_{2}$, DCPD and DCPA phase planes. Figure 2 illustrates the domain of chemical stability of HAp as a convex faceted hull with vertices $P_{i}$ (with i=1,…,11). These correspond to regions in phase space where three different phases coexists with HAp. Table S1 in Supplementary Materials contains the calculated values of chemical potentials at these intersection points. We note that there is no boundary with Ca metal, with the upper limit for $\mu_{\mathrm{Ca}}-\mu_{\mathrm{Ca}}^{0}$ being −1.8 eV. This means that if $\mu_{\mathrm{Ca}}=\mu_{\mathrm{Ca}}^{0}$ was assumed, formation energies of defects involving Ca substitutions would be affected by an error of ∼2 eV. In the analysis regarding substitutional iron defects, we will consider the following chemical phase space conditions:

1. Ca-rich and P-rich, points $P_{7}$ and $P_{8}$, $\mu_{\mathrm{Ca}}-\mu_{\mathrm{Ca}}^{0}=-1.8$ eV, $\mu_{P}-\mu_{P}^{0}=0$;
2. Ca-poor, point $P_{3}$, $\mu_{\mathrm{Ca}}-\mu_{\mathrm{Ca}}^{0}=-7.8$ eV, $\mu_{P}-\mu_{P}^{0}=-7.9$ eV;
3. P-poor, points $P_{1}$ and $P_{2}$, $\mu_{\mathrm{Ca}}-\mu_{\mathrm{Ca}}^{0}=-6.0$ eV, $\mu_{P}-\mu_{P}^{0}\approx -10.7$ eV.

### 3.2. Notation for Defect Structures

Before describing the structure of the iron defects let us first establish a few notation rules. We denote the defect structure as $M_{S}^{n}$, where *M* is a substituting species, most notably iron. However, this can also be a vacancy *V* or group of atoms (e.g., OH). *S* denotes the position of the defect, which can be a crystalline atomic site (Ca(I), Ca(II) or P) or an interstitial site (i(A), i(B), etc). The flipping of an hydroxyl unit is denoted as OH${}_{\mathrm{HO}}$. The number *n* is the formal oxidation state of iron, estimated from the occupation of the d-orbitals [49]. Table 2 shows the correspondence between the used notation, Kröger-Vink notation and the net charge of the defective supercell. We found a systematic correspondence between the formal oxidation state of iron atom and the defect charge state. This indicates that the gap states of the Fe defects under investigation (which can trap electrons or holes) belong to the 3d shell.

### 3.3. Neutral Iron Defects

We start by considering neutral iron defects based on structures previously proposed in the literature. Table 3 summarizes the relevant data collected. The first seven rows correspond to the Fe${}_{\mathrm{Ca}}$ substitutions, while the next five to iron interstitials. There is no apparent correlation between the defect structure and sample preparation conditions (e.g., Fe concentration or synthesis temperature).

**Figure 3:**
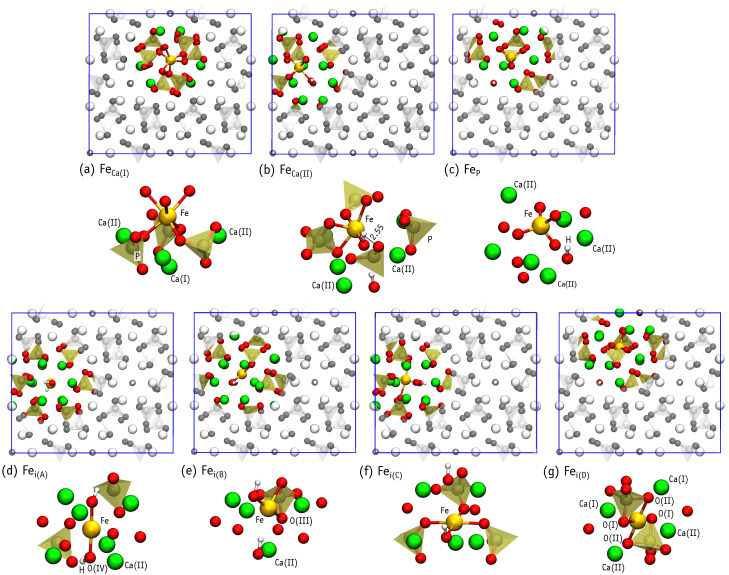
Illustration of neutral defect structures in Fe-HAp. For each defect, we show a top view of its location within supercell and a lateral view of its detailed atomic structure in the vicinity of ∼4 Å around the iron atom.

We considered Ca(I), Ca(II), as well as P substitutions, the later being unexplored in the literature. Figure 3a–c illustrate the substitutional defect structures obtained after relaxation, while Figure 3d–g depict the interstitial structures. The relaxation of the structure where iron was initially set up with the Fe${}_{i(A)}$ configuration (Wyckoff position 2b), resulted in another structure, where iron is displaced away from the center of the channel. This new structure is denoted as Fe${}_{i(C)}$ (Figure 3f) and approximately corresponds to Wyckoff position 12i. In order to achieve a stable Fe${}_{i(A)}$ configuration, one of the nearest OH groups has to be flipped (Figure 3d). Another interstitial position with the iron ion displaced from the center of the channel is labeled as Fe${}_{i(B)}$ (Figure 3e) and corresponds to Wyckoff position 2c. The Fe${}_{i(C)}$ structure may be slightly modified by flipping of the neighboring OH group, leading to a minute (∼0.1 eV) benefit in the total energy. Such small difference is lower than the flipping of isolated OH, estimated as 0.22 eV. Our result suggest that OH flipping may be stimulated by the presence of interstitial iron in the channel.

Finally, we considered iron inserted in the region between PO${}_{4}$ groups (Wyckoff position 6g), marked as Fe${}_{i(D)}$ (Figure 3g). At this location, the negatively charged PO${}_{4}^{3-}$ groups are expected to screen the positive charge of the iron ion. Although we scanned the relative stability of other structures, those whose formation energy was above 7 eV were discarded and not investigated further (e.g., Fe${}_{\mathrm{OH}}$ and Fe${}_{i}$ in the vicinity of the Ca(I) column).

Table 4 shows the formation energies of the most stable defects, estimated according to Equation (1) using chemical potentials at extreme points of the HAp stability diagram, namely for material grown under Ca- and P-rich ($P_{7}$ and $P_{8}$), Ca-poor ($P_{3}$) and P-poor ($P_{1}$) conditions. At Ca- and P-rich conditions, the ${\mathrm{Fe}}_{i(A)}^{0}$ defect is likely to be the most probable as it shows the lowest formation energy. Other stable defects (within less that 1 eV above the ground state) are ${\mathrm{Fe}}_{i(B)}^{0}$ and ${\mathrm{Fe}}_{\mathrm{Ca}}^{2+}$ substitutions. Other interstitial sites (${\mathrm{Fe}}_{i(C)}^{0}$, ${\mathrm{Fe}}_{i(D)}^{0}$) have higher formation energies. However, they still should be considered since hole or electron trapping may stabilize them.

At Ca-poor conditions ${\mathrm{Fe}}_{\mathrm{Ca}}^{2+}$ defects are the most stable, with formation energy ∼5 eV below that of ${\mathrm{Fe}}_{i(A)}^{0}$. A low value of $\mu_{P}$ at Ca-poor conditions ($P_{3}$ in Figure 2) leads to an easier depletion of phosphorus and to the stabilization of ${\mathrm{Fe}}_{P}^{5+}$ as well. Subsequently, at P-poor conditions this effect is further enhanced and ${\mathrm{Fe}}_{P}^{5+}$ becomes nearly 4 eV more stable than ${\mathrm{Fe}}_{\mathrm{Ca}}^{2+}$, and about 7.5 eV than the most favorable interstitial defect, ${\mathrm{Fe}}_{i(A)}^{0}$.

Table 4 reports the number of oxygen atoms neighboring iron (coordination number), their respective Fe-O distances, and the magnetic moment of each defect. The six-fold coordination of Fe${}_{\mathrm{Ca}(I)}$ (Figure 3a) is in line with the results of Jiang et al. [15], Low et al. [16] and Zilm et al. [17]. The five-fold coordinated Fe${}_{\mathrm{Ca}(\mathrm{II})}$ structure differs: six-fold coordinated iron is proposed by Jiang et al, while four-fold – by Zilm et al. However, we may count six neighbors if we include the far oxygen neighbor at distance $R_{\mathrm{Fe}-O}=2.55$ Å marked with dashed line on Figure 3b. Zilm et al obtained two oxygen atoms at high distances of $R_{\mathrm{Fe}-O}=2.73$ and 2.97 Å, but excluded them from the neighbors count, resulting in four-fold coordination.

**Figure 4:**
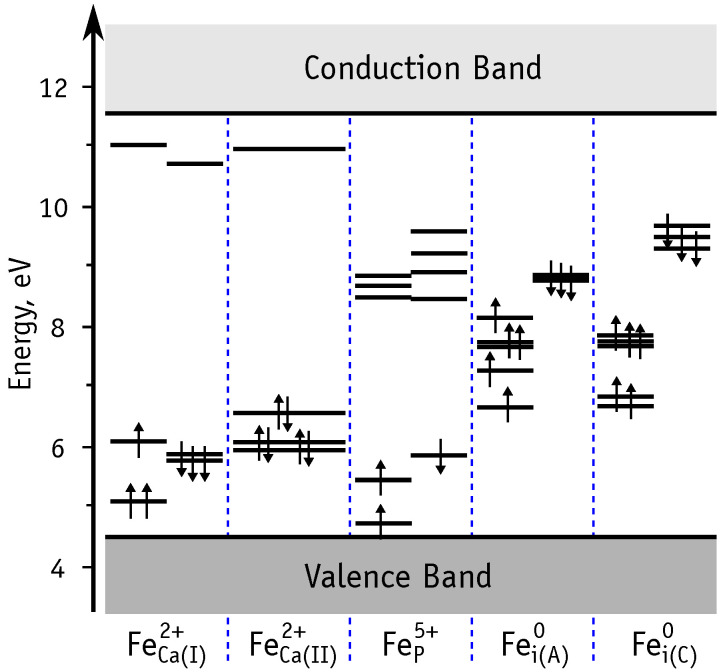
Energy level diagram based on the Khon-Sham states of Fe defects in HAp.

The structure of Fe${}_{i(A)}$ with two O(IV) neighbors shows Fe-O distances ∼0.2 Å longer than those observed by XRD [18]. This may result from the XRD analysis, which gives an ordered structure where oxygen atoms are fixed to crystallographic sites, or may reflect the distances of a positively charged state, where the iron cation is closer to the oxygen anions. Fe${}_{i(C)}$ configurations have four-fold coordinated iron. This is at variance with the XRD study of Gomes et al. [18], where three-fold coordinated iron was found, but is in line with XRD results of Kato et al. [19]. The only three-fold coordination of Fe that we find is in the Fe${}_{i(B)}$ structure (Figure 4e). However, the geometry differs from that proposed in Ref. [18], where Fe connects to one oxygen atom from a neighboring PO${}_{4}$ group and two O atoms from OH groups. In general, the calculated Fe-O distances look slightly overestimated. Of course, the picture could improve when considering charged cells.

### 3.4. Charged Iron Defects

Iron (Fe:4s${}^{2}$3d${}^{6}$) is not isoelectronic with respect to the species being replaced (Ca:4s${}^{2}$ and P:3s${}^{2}$3p${}^{3}$). Hence, Fe impurities are expected to create states within the band gap of HAp. These states are localized and may act as hole or electron traps. The description of the Fe-HAp requires the consideration these cases, which can be accounted for by changing the occupation of the highest occupied gap states of the defective supercell. In a real crystal, the capture of a hole (creation of a local positive charge) can be compensated by numerous possibilities: cation vacancies $V_{\mathrm{Ca}}^{-}$, $V_{H}^{-}$, foreign anion interstitials like [CO${}_{3}^{2-}$]${}_{i}$, etc., thus leading to a lowering of the Fermi energy (electron chemical potential), We leave the exact mechanisms of charge compensation out of the scope of this work.

An qualitative picture of the charge states allowed for each defect can be found from the respective Kohn-Sham levels in the band gap [61]. Positive charge states (hole trapping) requires the presence of filled states in the gap, while the negatively charged defects (electron trapping) requires the presence of empty states. Figure 4 illustrates the energy of the one-electron states in the band gap obtained from spin-polarized calculations of substitutional and interstitial defects in the neutral charge state.

Substitutional iron on Ca sites, Fe${}_{\mathrm{Ca}}^{2+}$, have six electrons in the 3d shell, but after the first ionization all filled states move below the valence band top. Hence, further ionization would require an energy equivalent to the band gap width (∼7 eV), which essentially tell us that Fe${}_{\mathrm{Ca}}^{2+}$ defects can be single donors, but not double donors. Unoccupied states of Fe${}_{\mathrm{Ca}}^{2+}$ are rather close to the conduction band, suggesting that they are not acceptors either (can not trap electrons).

Iron on the phosphorous site in the Fe${}_{P}^{5+}$ state (neutral charge state) has three filled one-electron states and 7 empty states in the gap (3d${}^{3}$ configuration). We will show below that Fe${}_{P}^{5+}$ can trap one hole or up to two electrons, becoming Fe${}_{P}^{6+}$ or Fe${}_{P}^{4+}$ and Fe${}_{P}^{3+}$ states, respectively.

Neutral interstitial defects show only filled states in the gap. These can trap up to three holes, thus leading to Fe${}_{i}^{+}$, Fe${}_{i}^{2+}$ and Fe${}_{i}^{3+}$, respectively.

**Figure 5:**
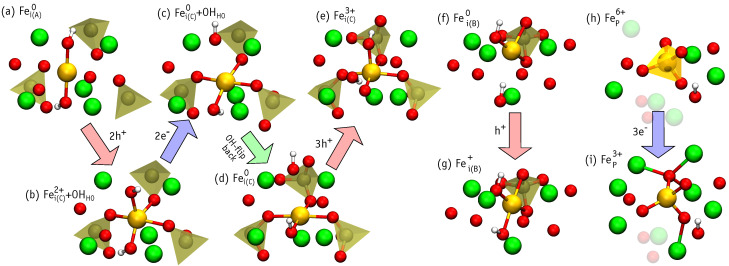
Illustration of the change in the local atomic structure of selected iron defects (ligands within ≲4 Å from the Fe atom are also depicted), following the capture of electrons or holes.

We found that the structure of the defects depend strongly on the local charge. Figure 5 illustrates some of the most remarkable structural changes of the defects induced by the capture of electrons and holes. Additional reconfigurations are depicted in Figure S1 in Supplementary Information. Table 5 presents the number of nearest oxygen neighbors to the Fe ion ($N_{\mathrm{Fe}-O}$), the range of Fe-O bond lengths ($R_{\mathrm{Fe}-O}$), as well as the magnetic moment of the defect ($M_{z}$). Note, that some states exhibit a high magnetization, up to $5\mu_{B}$ for Fe${}_{\mathrm{Ca}(I)}^{3+}$ and Fe${}_{i(C)}^{3+}$. These are to be compared to the case of neutral defects, where the magnetic moment was at most $2\mu_{B}$.

In general, an increase of the positive charge leads to a decrease of Fe-O bond lengths. This effect is strikingly illustrated in Figure 5h,i, which showcase the Fe${}_{P}^{3+}$–Fe${}_{P}^{6+}$ sequence of defects. This rule is understandably violated when there is a change in the coordination of the Fe ion, and therefore, a significant modification of the local electrostatics. Examples are the increases of $R_{\mathrm{Fe}-O}$ in ${\mathrm{Fe}}_{i(B)}^{0}$, ${\mathrm{Fe}}_{i(C)}^{+}$, or ${\mathrm{Fe}}_{i(D)}^{2+}$ upon hole capture,

${\mathrm{Fe}}_{i(B)}^{0}+h^{+}\to {\mathrm{Fe}}_{i(B)}^{+}$ (Figure 5f,g),

${\mathrm{Fe}}_{i(C)}^{+}+h^{+}\to {\mathrm{Fe}}_{i(C)}^{2+}$ (Figure S1p,q),

${\mathrm{Fe}}_{i(D)}^{2+}+h^{+}\to {\mathrm{Fe}}_{i(D)}^{3+}$ (Figure S1y,z).

The above processes are reversible, i.e., electron trapping at (or hole emission from) the positively charged defects result in the recovery of the longer bond lengths. As expected, HAp cations repel the iron ion. In the case of ${\mathrm{Fe}}_{i(C)}^{3+}$ without OH flipping, this effect leads to migration of a proton from OH to a close PO${}_{4}$ group (see Figure 5d,e). We found that the high-symmetry defect configuration ${\mathrm{Fe}}_{i(A)}^{2+}$ is not stable and spontaneously transforms to ${\mathrm{Fe}}_{i(C)}^{2+}$. Hence, upon hole capture by (or electron emission from) ${\mathrm{Fe}}_{i(A)}^{+}$, the iron moves toward the edge of the OH channel (see Figure 5a,b),

$${\mathrm{Fe}}_{i(A)}^{+}+h^{+}\to {\mathrm{Fe}}_{i(C)}^{2+}+{\mathrm{OH}}_{\mathrm{HO}}.$$

The reverse process involves overcoming a barrier (not calculated), and ${\mathrm{Fe}}_{i(A)}^{+}$ is not recovered spontaneously (see Figure 5b,c).

**Figure 6:**
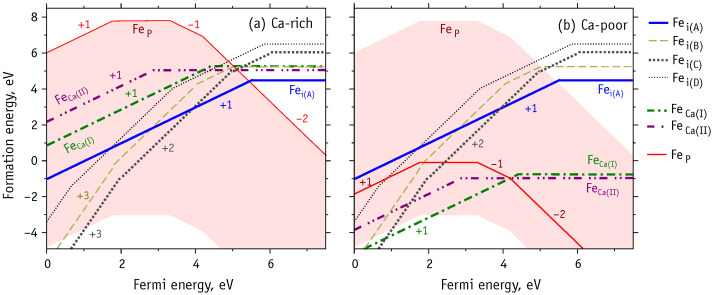
Formation energies of Fe-HAp as a function of the Fermi energy at extreme points of HAp stability diagram: (**a**) Ca-rich and P-rich, (**b**) Ca-poor. The horizontal segments correspond to neutral simulation cells (Table 4), and slanted – to non-neutral with charge marked by numbers from −2 to +3.

The formation energy of a charged defect is a function of the Fermi energy (c.f. Equation (1)). This dependence is clearly illustrated in Figure 6a,b, which show the results for Ca- and P-rich material and for Ca-poor HAp, respectively. The red shadow area on both diagrams indicates the whole range for the formation energy of Fe${}_{P}$. The upper bound corresponds to P-rich conditions, while the lower bound to P-poor material. The solid red line in Figure 6b shows the formation energy of Fe${}_{P}$ under Ca-poor conditions. Thick dashed-dotted lines correspond to formation energies of Fe${}_{\mathrm{Ca}}$ defects, and thick solid line and other non-solid lines correspond to interstitial defects (see legend).

According to our results, the phosphorous substitutions, especially Fe${}_{P}^{3+}$ (charge state q=−2) is a rather stable species when there is abundance of electrons in the material (n-type HAp). This is even more evidenced in Ca-poor and P-poor conditions, where in p-type material we expect Fe${}_{P}^{4+}$, Fe${}_{P}^{5+}$ and even Fe${}_{P}^{6+}$ to become more stable and compete with other species, namely Fe${}_{i}$ and Fe${}_{\mathrm{Ca}}$.

However, the phosphorous substitutions were not previously considered in the literature, since the replacing of phosphorus by iron cation requires the breaking of pretty stable P–O bonds of PO${}_{4}$ group. Alternatively, the replacement of whole PO${}_{4}$ group by FeO${}_{4}$ one will provide the same structural result. The obtained most probable Fe${}_{P}$ configuration is tetrahedral coordinated ferric cation ([FeO${}_{4}{]}^{5-}$ group) is not unusual and can be found in magnetite [52] or in iron-phosphate glass [62].

Regarding substitutional Fe at calcium sites, we find that Fe${}_{\mathrm{Ca}(I)}^{3+}$ is the most probable form under Ca-poor and p-type conditions (Figure 6b). We note that despite Fe${}_{\mathrm{Ca}(\mathrm{II})}^{2+}$ having lower formation energy than Fe${}_{\mathrm{Ca}(I)}^{2+}$ in n-type HAp, the Fe${}_{P}^{3+}$ species is even more stable, thus making the phosphorus substitution more probable than a replacement of calcium.

In contrast, under Ca- and P-rich conditions (Figure 6a) the interstitial defects are expected to prevail, especially in p-type and intrinsic HAp. Depending on the Fermi level location, the most probable states are Fe${}_{i(C)}^{3+}$, Fe${}_{i(C)}^{2+}$ and Fe${}_{i(A)}^{+}$ defects. The two-fold coordinated Fe${}_{i(A)}^{0}$ defect, is more favorable in n-type HAp, yet again, Fe${}_{P}^{3+}$ is more stable and more likely to form.

We can compare the obtained local atomic structure of iron defects in HAp (see Table 4 and Table 5) with those reported in the literature (Table 3). Iron substitutions with long distances $R_{\mathrm{Fe}-O}\geq 2.2$ Å and 6 oxygen neighbors [15,16] are best described by Fe${}_{\mathrm{Ca}(I)}^{2+}$. The slightly more favorable Fe${}_{\mathrm{Ca}(\mathrm{II})}^{2+}$ substitution has 5 oxygen neighbors, which could explain the result of Boda et al. [6]. The iron with only two neighbors in the study of Gomes et al. [18] could be described by ${\mathrm{Fe}}_{i(A)}^{+}$, although the small Fe-O distances of 1.7 Å are only reproduced by Fe${}_{P}^{6+}$, which is rather unstable. Iron with three [18] or four [19] oxygen neighbors at distances $R_{\mathrm{Fe}-O}\geq 1.8$ Å can be accounted for by ${\mathrm{Fe}}_{i(C)}^{3+}$ or Fe${}_{P}^{3+}$ defects.

In summary, we find that Fe-HAp can contain both substitutional and interstitial defects depending on the preparation conditions. The phosphorus substitutions have iron in low-spin states ($M_{z}\leq \mu_{B}$,), making them less useful for many applications envisaged for Fe-HAp. To avoid those defects the synthesis should be performed closer to P-rich and p-type conditions. In that case we expect the formation of high-spin defects with $M_{z}=5\mu_{B}$ (Fe${}_{\mathrm{Ca}(I)}^{3+}$ and Fe${}_{i(C)}^{3+}$) and $M_{z}=4\mu_{B}$ (Fe${}_{i(C)}^{2+}$).

### 3.5. Fe K-XANES of Fe-HAp

The near-edge structure of X-ray absorption (XANES) spectra is particularly sensitive to the details of local atomic structure of the absorbing element [51]. X-ray absorption spectroscopy has been applied to materials without long range order, and that includes the Fe-HAp. We consider the experimental spectra of Fe-HAp published by Gomes et al. [18]. We also keep the notation of the original study regarding the experimental conditions, i.e., 15Fe-500 and 15Fe-1100 which correspond to 15 mol % of Fe per HAp unit cell of samples sintered at 500 °C and 1100 °C, respectively.

**Figure 7:**
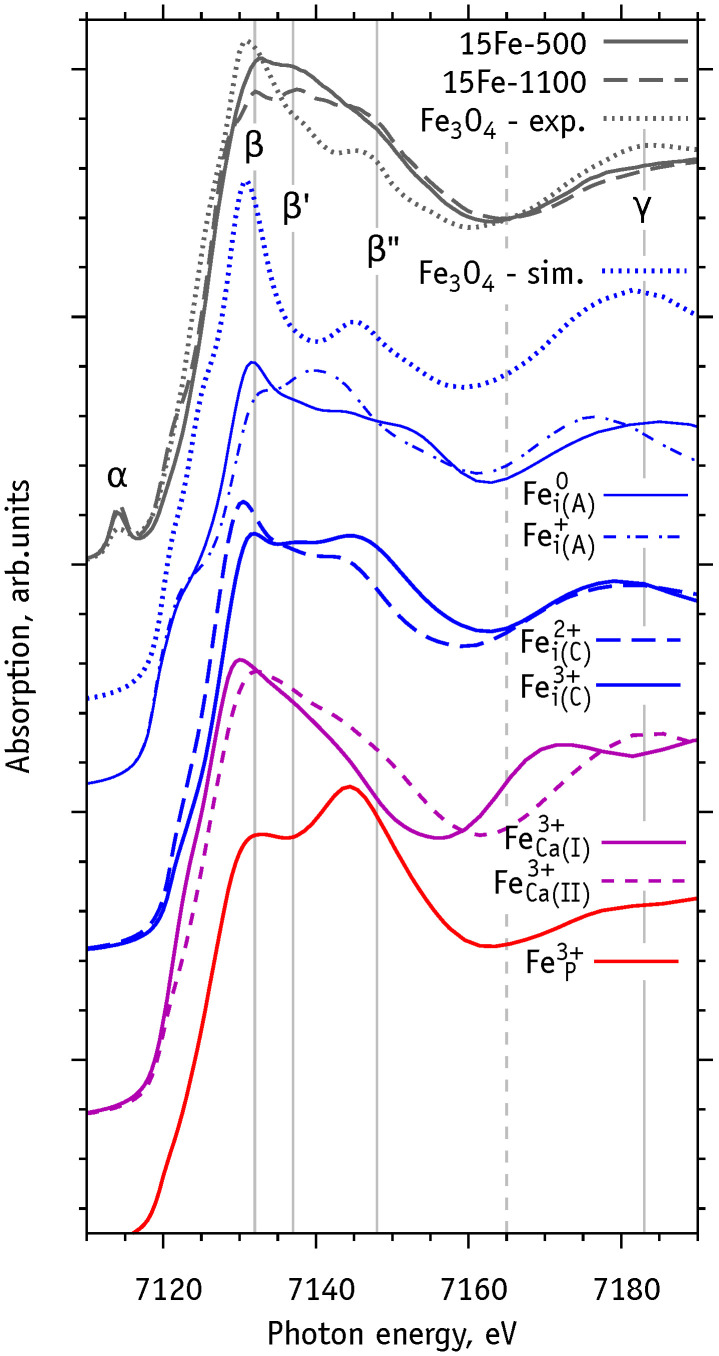
Comparison between experimental Fe K-XANES spectra of Fe-HAp published in Ref. [18] (top three curves) and simulated spectra (all other curves) as described in the text.

The top three (black colored) curves in Figure 7 shows the experimental data of Gomes et al. [18] for Fe-HAp and for magnetite (Fe${}_{3}$O${}_{4}$). The latter was used as a reference for the alignment of the theoretical energy scale to the experimental one. Greek letters mark the main spectral features: pre-edge (α), the main peak (β), its satellite peaks ($\beta^{\prime }$ and $\beta^{\prime \prime }$), and a more distant peak (γ). The vertical dashed line in Figure 7 provides guidance for the relative positions of the minimum between features β and γ.

In order to determine the types of Fe defects in the 15Fe-500 and 15Fe-1100 samples we simulated the Fe K-XANES spectra for each structure considered in Section 3.3 and Section 3.4. Figure 7 shows the simulated spectra of the most probable defect structures. Figure S2 in Supplementary Information shows all calculated spectra. The comparison of experimental and simulated spectra for magnetite Fe${}_{3}$O${}_{4}$ (dotted curves in Figure 7) reveals the main insufficiencies of the simulation method, including (i) the lack of pre-edge features (α) and (ii) a poor reproduction of the $\beta^{\prime }$ satellite. However, the intensities and energy positions of the main features (β, $\beta^{\prime \prime }$ and γ) of the magnetite spectrum are correctly reproduced. All spectral features of another reference iron oxide, hematite Fe${}_{2}$O${}_{3}$, could be reproduced (not shown) using exact the same calculation scheme. In this case, the differences between experimental and simulated spectra show qualitatively the same insufficiencies as for magnetite.

The simulated spectra for Fe-HAp show good sensitivity with respect to their atomistic structures and charge states. In most cases the increase of the iron oxidation state shifts the position of the main peak (β) to higher energies. This follows from an increase of the local positive charge and binding energy of K-electrons. An exception is seen for the Fe${}_{i(D)}^{3+}$ spectrum (Figure S2d), which is explained by significant changes in its local atomic structure (coordination change from 4 to 6) upon ionization.

The relative intensities $I_{\beta }$ and $I_{\beta^{\prime \prime }}$, corresponding to features β and $\beta^{\prime \prime }$ from the simulated spectra, are sensitive to the defect type: phosphorus substitutions have $I_{\beta }<I_{\beta^{\prime \prime }}$, calcium substitutions show $I_{\beta }>I_{\beta^{\prime \prime }}$, and most interstitial defects have $I_{\beta }∼I_{\beta^{\prime \prime }}$. Additionally, the spectra show considerable differences concerning the position and shape of the γ feature.

The spectrum of the 15Fe-1100 sample shows comparable β and $\beta^{\prime \prime }$ intensities ($I_{\beta }∼I_{\beta^{\prime \prime }}$), suggesting that most Fe defects are of interstitial character. Conversely, the higher intensity of peak β in the spectrum of sample 15Fe-500 may indicate the presence of substitutional Fe on calcium sites or ${\mathrm{Fe}}_{i(A)}^{n}$ defect (n=0, 1+). The latter scenario is in line with the conclusions of Gomes et al. [18], where the formation of the high-symmetry Fe${}_{i(A)}$ structure was proposed in the 15Fe-500 and 15Fe-800 samples, whereas Fe${}_{i(C)}$-type defects were suggested for the high-temperature treated 15Fe-1100 sample.

## 4. Conclusions

We performed a hybrid-DFT study of iron defects in hydroxyapatite. The most favorable structures (and respective charge states) were used to simulate Fe K-XANES spectra of Fe-HAp. These were compared to experimental data available in the literature. The results allow us to draw the following conclusions:

1. The chemical stability of HAp (Ca${}_{10}$(PO${}_{4}{)}_{6}($OH${)}_{2}$) is limited by a thermodynamic equilibrium with O${}_{2}$, CaO, Ca(OH)${}_{2}$, H${}_{2}$, P, DCPD (CaHPO${}_{4}\cdot 2$H${}_{2}$O), DCPA (CaHPO${}_{4}$) and TCP (Ca${}_{3}$(PO${}_{4}{)}_{2}$) phases. Formation of these compounds limit the range of values of Ca and P chemical potentials, and we could identify three extreme cases: Ca- and P-rich, Ca-poor, P-poor.
2. Under P-poor conditions, phosphorous substitutions are the most favorable, resulting in the formation of low-spin Fe defect states. However, p-type HAp may contain high spin iron interstitials Fe${}_{i(C)}^{3+}$.
3. Under Ca-poor conditions, the high-spin calcium substitution, Fe${}_{\mathrm{Ca}(I)}^{3+}$, is the most favorable species. Fe${}_{\mathrm{Ca}}^{2+}$ defects have higher formation energy comparing to Fe${}_{P}^{3+}$ and Fe${}_{\mathrm{Ca}(I)}^{3+}$.
4. Under Ca- and P-rich conditions, interstitial iron atoms in the OH channel are the most prominent. Depending on the position of Fermi level the most favorable are Fe${}_{i(C)}^{3+}$, Fe${}_{i(C)}^{2+}$ and Fe${}_{i(A)}^{+}$. When compared to Fe${}_{i(C)}$, the last structure involves the flipping of a nearby hydroxyl unit. The OH flipping does not introduce a significant change to the formation energy of Fe${}_{i(C)}^{3+}$ and Fe${}_{i(C)}^{2+}$ defects.
5. High-spin iron defects are Fe${}_{\mathrm{Ca}(I)}^{3+}$ and Fe${}_{i(C)}^{3+}$. These are both expected in p-type HAp. Such configurations are expected to be most useful for materials targeting magnetic hyperthermia or magnetic resonance imaging applications.
6. The comparison of Fe K-XANES spectra of theoretically predicted defect structures with experimental data [18] confirms the interstitial character of iron defects in samples sintered at high (1100 °C) temperature, but does not exclude the substitution defects for samples sintered at lower temperatures.

## Figures and Tables

**Table 1:** Comparison of calculated heats of formation $\Delta H_{f}$ (eV/formula unit), cell volumes Ω (Å${}^{3}$) and bulk modulus *B* (GPa) of compounds with available reference data [53,54,55,56,57].

Compound		HSE				Ref.	
	$\Delta H_{f}$	Ω	B		$\Delta H_{f}$	Ω	B
HAp	−64.40	532.1	87		−69.44	528.7	89
CaO	−6.00	110.4	114		−6.58	110.5	116
Ca(OH)${}_{2}$	−9.63	56.46	31		−10.23	54.78	33
P${}_{2}$O${}_{5}$	−14.22	330.7	64		−15.60	322.0	12–40
H${}_{3}$PO${}_{4}$	−11.52	–	–		−13.35	–	–
H${}_{2}$O	−2.61	–	–		−2.97	–	–
TCP	−39.2	445.5	105		−42.8	445.5	100
TTCP	−45.12	796.0	79		−49.4	797.3	–
DCPD	−23.89	490.3	51		−22.33	497.7	–
DCPA	−17.41	311.9	75		–	309.3	–

**Table 2:** Correspondence between the defect charge state *q* (in units of *e*), Kröger-Vink notation and that used in this work (right-most column).

	Notation	
*q*	Kröger-Vink	This Work
0	${\mathrm{Fe}}_{i}^{\times }$	${\mathrm{Fe}}_{i}^{0}$
+1	${\mathrm{Fe}}_{i}^{•}$	${\mathrm{Fe}}_{i}^{+}$
+2	${\mathrm{Fe}}_{i}^{••}$	${\mathrm{Fe}}_{i}^{2+}$
+3	${\mathrm{Fe}}_{i}^{•••}$	${\mathrm{Fe}}_{i}^{3+}$
0	${\mathrm{Fe}}_{\mathrm{Ca}}^{\times }$	${\mathrm{Fe}}_{\mathrm{Ca}}^{2+}$
+1	${\mathrm{Fe}}_{\mathrm{Ca}}^{•}$	${\mathrm{Fe}}_{\mathrm{Ca}}^{3+}$
−2	${\mathrm{Fe}}_{P}^{\prime \prime }$	${\mathrm{Fe}}_{P}^{3+}$
−1	${\mathrm{Fe}}_{P}^{\prime }$	${\mathrm{Fe}}_{P}^{4+}$
0	${\mathrm{Fe}}_{P}^{\times }$	${\mathrm{Fe}}_{P}^{5+}$
+1	${\mathrm{Fe}}_{P}^{•}$	${\mathrm{Fe}}_{P}^{6+}$

**Table 3:** List of proposed structures for Fe defects in Fe-HAp. Also included (when available) are the iron-oxygen interatomic distances $R_{\mathrm{Fe}-O}$, numbers of oxygen neighbors $N_{\mathrm{Fe}-O}$, number of Fe atoms per HAp unit cell $n_{\mathrm{Fe}}$, temperatures of sample preparation (doping treatment or sintering) and detection methods.

Structure	$R_{\mathrm{Fe}-O}$, Å	$N_{\mathrm{Fe}-O}$	$n_{\mathrm{Fe}}$	$T_{\mathrm{prep}.}$, ${}^{{}^{\circ}}$C	Methods	Ref.
Fe${}_{\mathrm{Ca}(I)}$				1000	MB	[14]
Fe${}_{\mathrm{Ca}(\mathrm{II})}$			≥0.15		XRD	[58]
Fe${}_{\mathrm{Ca}(I)}$	2.2–2.3	6	0.5	90	MD, EPR, MB	[15]
Fe${}_{\mathrm{Ca}}$	2.4–2.5	6	≤0.5	600–1000	EPR, MB	[16]
Fe${}_{\mathrm{Ca}}$			0.2	40	XRD, XAS	[9]
Fe${}_{\mathrm{Ca}(\mathrm{II})}$/Fe${}_{\mathrm{Ca}(I)}$		5 or 6/6	0.5–2.0	950	XRD, XPS, MB	[6]
Fe${}_{\mathrm{Ca}(\mathrm{II})}$/Fe${}_{\mathrm{Ca}(I)}$	2.0–2.2/2.2–2.3	4 / 6	0.012	25	XRD, DFT	[17]
Fe${}_{\mathrm{Ca}(\mathrm{II})}$			0.3–6.0	100	XPS, XRD	[59]
FeOOH				biogenic/120	MB	[5,60]
Fe${}_{i(A)}$	1.7	2	0.15–0.75	<1000	XRD	[18]
Fe${}_{i(C)}$	1.80–1.85	3	0.15–0.75	1100	XRD	[18]
Fe${}_{i(C)}$	1.84–1.94	3±0.7	0.15	1100	EXAFS	[18]
Fe${}_{i(C)}$	1.8–2.4	4	0.1–0.9	60	XRD, IR	[19]

**Table 4:** Formation energies ($E_{f}$ in eV) under extreme growth conditions of HAp, number of nearest oxygen neighbors ($N_{\mathrm{Fe}-O}$), Fe-O bond length ($R_{\mathrm{Fe}-O}$ in Å) and magnetic moment ($M_{z}$ in $\mu_{B}$ units) calculated for several defect configurations in the neutral charge state, q=0.

Defect Structure		$E_{f}$		$N_{\mathrm{Fe}-O}$	$R_{\mathrm{Fe}-O}$		$M_{z}$
	Ca- & P-Rich	Ca-Poor	P-Poor				
${\mathrm{Fe}}_{\mathrm{Ca}\left(I\right)}^{2+}$	5.25	−0.76	1.02	6	2.04–2.24		0.0
${\mathrm{Fe}}_{\mathrm{Ca}\left(\mathrm{II}\right)}^{2+}$	5.05	**−0.96**	0.82	5	1.99–2.21		0.0
${\mathrm{Fe}}_{P}^{5+}$	6.05	−0.09	**−3.03**	4	1.69–1.70		1.0
${\mathrm{Fe}}_{i(A)}^{0}$	**4.48**	4.48	4.48	2	1.88–1.89		2.0
${\mathrm{Fe}}_{i(B)}^{0}$	5.24	5.24	5.24	3	1.86–2.22		2.0
${\mathrm{Fe}}_{i(C)}^{0}$	6.05	6.05	6.05	4	1.94–2.18		2.0
${\mathrm{Fe}}_{i(D)}^{0}$	6.50	6.50	6.50	4	2.02–2.06		2.0

**Table 5:** Charge state, *q*, Number of oxygen neighbors, $N_{\mathrm{Fe}-O}$, and respective range of distances, $R_{\mathrm{Fe}-O}$ (Å), and magnetization $M_{z}$ ($\mu_{B}$ units) calculated for charged iron defects in HAp.

Defect Structure	*q*	$N_{\mathrm{Fe}-O}$	$R_{\mathrm{Fe}-O}$		$M_{z}$
${\mathrm{Fe}}_{\mathrm{Ca}\left(I\right)}^{3+}$	+1	6	1.96–2.12		5.0
${\mathrm{Fe}}_{\mathrm{Ca}\left(\mathrm{II}\right)}^{3+}$	+1	5	1.90–2.01		1.0
${\mathrm{Fe}}_{P}^{6+}$	+1	4	1.65–1.67		0.0
${\mathrm{Fe}}_{P}^{4+}$	−1	4	1.74–1.75		0.0
${\mathrm{Fe}}_{P}^{3+}$	−2	4	1.81–1.84		1.0
${\mathrm{Fe}}_{i(A)}^{+}$	+1	2	1.84–1.85		3.0
${\mathrm{Fe}}_{i(B)}^{+}$	+1	4	1.89–2.02		3.0
${\mathrm{Fe}}_{i(B)}^{2+}$	+2	4	1.82–1.95		2.0
${\mathrm{Fe}}_{i(B)}^{3+}$	+3	4	1.78–1.88		3.0
${\mathrm{Fe}}_{i(C)}^{+}$	+1	4	1.89–2.08		3.0
${\mathrm{Fe}}_{i(C)}^{2+}$	+2	5	1.84–2.16		4.0
${\mathrm{Fe}}_{i(C)}^{3+}$	+3	5	1.72–2.12		5.0
${\mathrm{Fe}}_{i(D)}^{+}$	+1	4	1.98–1.99		3.0
${\mathrm{Fe}}_{i(D)}^{2+}$	+2	4	1.90–1.93		2.0
${\mathrm{Fe}}_{i(D)}^{3+}$	+3	6	1.95–2.06		1.0

## Data Availability

The atomic coordinates of optimized Fe-HAp structures are available in POSCAR format through Supplementary Materials.

## Supplementary Materials

The following are available online at https://www.mdpi.com/article/10.3390/nano11112978/s1, Table S1: Chemical potentials in eV at selected points from the phase stability diagram, Figure S1: Illustration of the local atomic structure of iron atom (≲4 Å) for all considered Fe-HAp configurations, Figure S2: Simulated Fe K-XANES spectra for the considered Fe-HAp structures. Pseudopotential files, example input script and quantum espresso output file. Atomic coordinates of Fe-HAp structures in POSCAR format.

## Abbreviations

The following abbreviations are used in this manuscript:

HAp	Hydroxyapatite
Fe-HAp	Iron-doped HAp
DFT	Density functional theory
HSE	Heyd, Scuseria and Ernzerhof / Hybrid Screened Exchange
XANES	X-ray absorption near-edge structure
