# Supplementary material for: Iron in Hydroxyapatite: Interstitial or Substitution Sites?
Source: Nanomaterials (Basel). 2021 Nov 5;11(11):2978. doi: 10.3390/nano11112978 (PMC8625999; doi:10.3390/nano11112978)
Supplement: Supplementary file 1 [file nanomaterials-11-02978-s001.zip › supplement/tabS1_figS1_figS2.pdf]

Iron in Hydroxyapatite: Interstitial or Substitution Sites?

Leon Avakyan
Southern Federal University, 5 Zorge St., Rostov-on-Don 344090, Russian Federation

Ekaterina Paramonova
Institute of Mathematical Problems of Biology, Keldysh Institute of Applied Mathematics, Russian Academy of Sciences, 1 Vitkevicha St., Pushchino, 142290, Moscow region, Russian Federation

Vladimir Bystrov
Institute of Mathematical Problems of Biology, Keldysh Institute of Applied Mathematics, Russian Academy of Sciences, 1 Vitkevicha St., Pushchino, 142290, Moscow region, Russian Federation

Sandrine Gomes
Université Clermont Auvergne, Clermont Auvergne INP, CNRS, ICCF, F-63000 Clermont-Ferrand, France

Guillaume Renaudin
Université Clermont Auvergne, Clermont Auvergne INP, CNRS, ICCF, F-63000 Clermont-Ferrand, France

SUPPLEMENTARY INFORMATION

Table S1. Chemical potentials in eV at selected points from the phase stability diagram (Figure 2 of main text).

P_i	$\mu_{\mathrm{Ca}} - \mu^0_{\mathrm{Ca}}$	$\mu_{\mathrm{P}} - \mu^0_{\mathrm{P}}$	$\mu_{\mathrm{O}} - \mu^0_{\mathrm{O}}$	$\mu_{\mathrm{H}} - \mu^0_{\mathrm{H}}$	**Coexisting phases**
P_1	−6.0	−10.8	0.0	−1.8	O_2, $Ca(OH)_2$, CaO
P_2	−6.0	−10.5	0.0	−2.6	O_2, CaO, TCP
P_3	−7.8	−7.9	0.0	−1.7	O_2, DCPA, TCP
P_4	−7.7	−8.1	0.0	−1.6	O_2, DCPA, DCPD
P_5	−6.7	−9.8	0.0	−1.5	O_2, $Ca(OH)_2$, DCPD
P_6	−2.4	−1.8	−3.6	0.0	H_2, $Ca(OH)_2$, CaO
P_7	−1.8	0.0	−4.3	0.0	H_2, CaO, P
P_8	−1.8	0.0	−4.2	−0.5	CaO, P, TCP
P_9	−4.7	0.0	−3.1	−0.2	DCPA, P, TCP
P_{10}	−4.5	0.0	−3.2	0.0	H_2, DCPA, DCPD, P
P_{11}	−3.7	−2.5	−2.9	0.0	H_2, $Ca(OH)_2$, DCPD

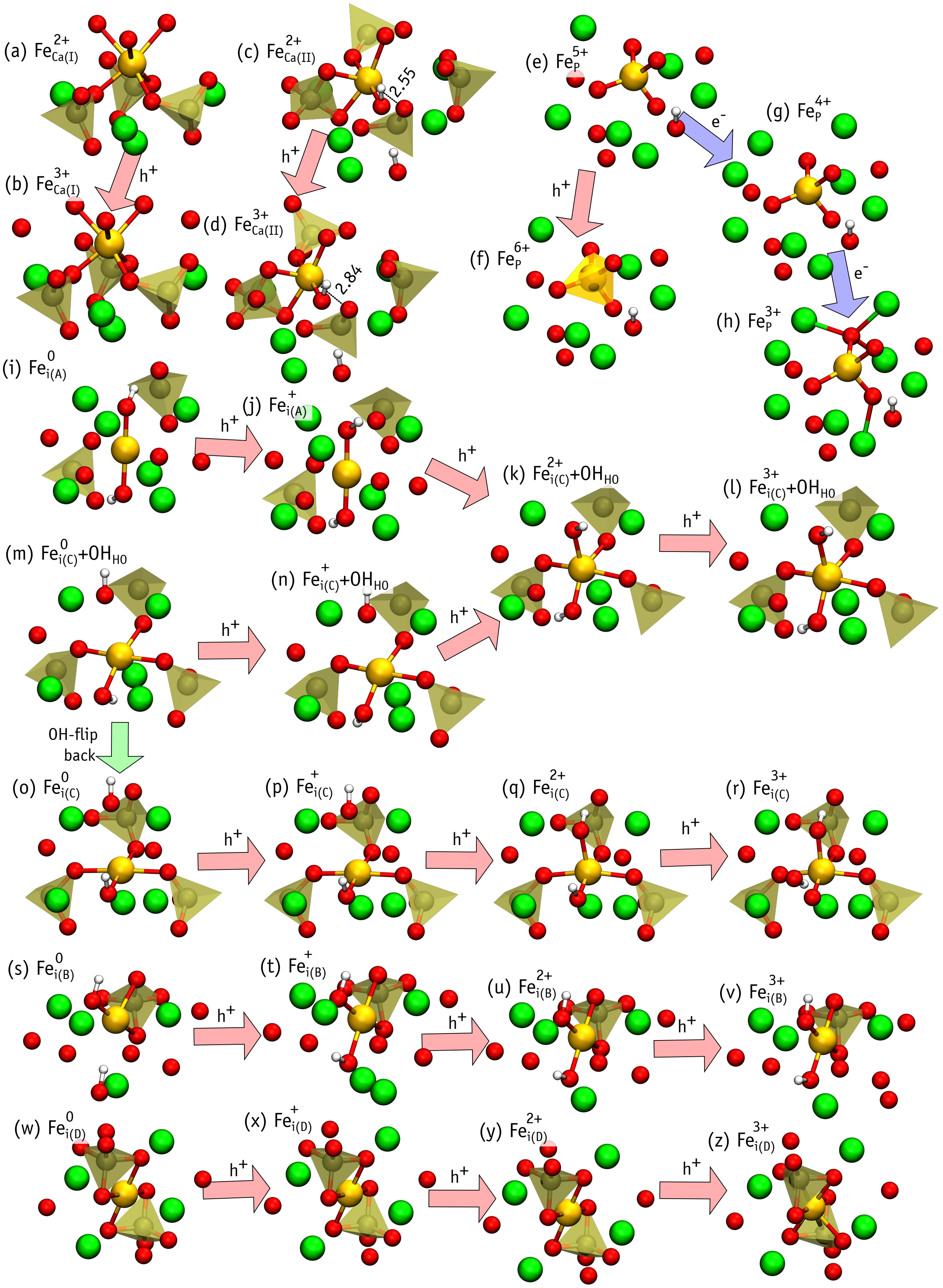

Figure S1. Illustration of the local atomic structure of iron atom ($\lesssim 4$ Å) for all considered Fe-HAp configurations. Iron, oxygen, calcium, hydrogen and phosphorus are shown in yellow, red, green, white, and inside the tetrahedra.

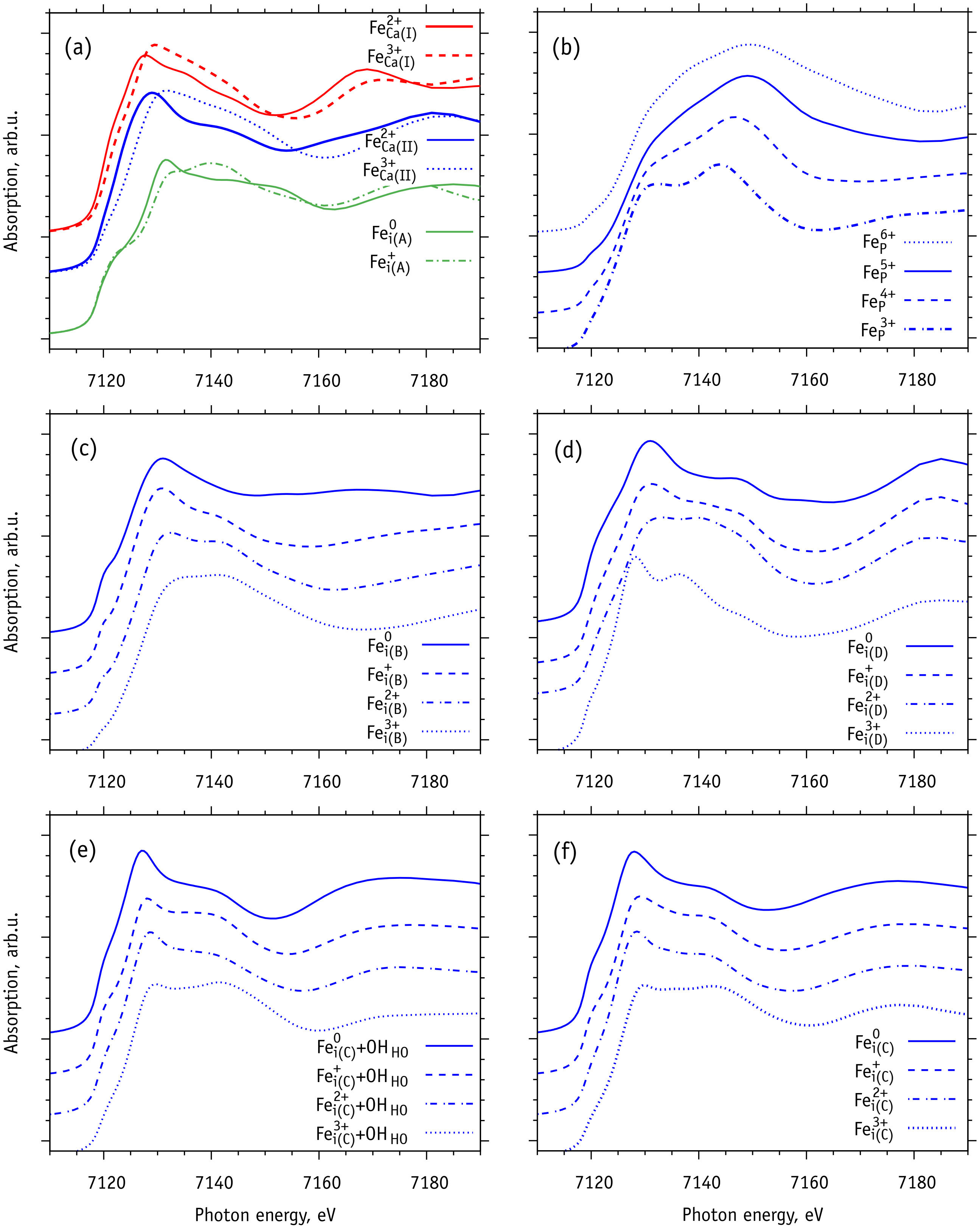

Figure S2. Simulated Fe K-XANES spectra for the considered Fe-HAp structures.
